# Temperature and AC electrical properties effects on phosphate natural mixture, Abu Tartur plateau, Western Desert, Egypt

**DOI:** 10.1038/s41598-025-09313-3

**Published:** 2025-07-31

**Authors:** Mohamed Mahmoud Gomaa

**Affiliations:** Geophysical Sciences Dept, National Research Centre, Geophysics Lab, Dokki, Cairo, Egypt

**Keywords:** Abu Tartur plateau, Phosphate, Conductivity, Dielectric constant, Temperature, Solid Earth sciences, Geophysics, Applied physics

## Abstract

The research is focused on examining electrical properties (conductivity and dielectric constant), mineralogy, and geochemical behaviors of natural phosphate mixtures, Abu Tartur plateau, Western Desert Egypt. Abu Tartur plateau has a rich supply of phosphorus deposits. Phosphate deposits mostly consist of fluorapatite (Ca_5_(PO_4_)_3_F). Our objective is to evaluate the changes in AC electrical properties, emphasizing the effects of temperature, and frequency variations. Electrical properties expand with temperature due to greater mobility of charge carriers at higher frequencies and at higher temperatures. Electrical characteristics were subjected to a temperature range of 60–700 ^0^C and frequencies of 42 Hz–5 MHz. Electrical properties of phosphate mixtures have a non-linear association with temperature and are highly dependent on frequency. As temperature rises, the conductivity of the mixture also increases, as evidenced by the temperature coefficient. The completion of these investigations will lead to a greater understanding of the geological and geochemical processes that lead to phosphate formation deposits, as well as the development of more effective industrial methods and material attributes. Temperature and conductivity are connected because dissolved ions increase both temperature and conductivity. Both conductivity and temperature are influenced by dissolved ions, hence they are coupled. Heterogeneity fluctuation can have a significant impact on the electrical characteristics of materials. Due to heterogeneity, the change in electrical characteristics is not monotonically affected by increasing conductor concentration. Presence of electrical features becomes more noticeable as temperature concentration increases. AC electrical conductivity of a phosphate natural combination from Abu Tartur and its fluctuation with temperature is inadequate and this work seeks to close this information gap.

## Introduction

AC electrical properties of natural phosphate resources (Abu Tartur plateau) and its variation with heat are poor and this work pursues to close this information gap. Abu Tartur plateau Western Desert’s has abundant phosphate resources that are used for fertilizers manufacturing and other industrial goods Abdeen & El-Naggar^1^.

Commercially significant concentrations of phosphate minerals reflect the complex depositional history of sedimentary strata, which was affected by maritime transgressions and regressions. The mineralogical investigation includes the identification of related gangue minerals, like carbonates and silicates, and primary phosphate minerals, like francolite [(Ca,Mg,Sr,Na)_10_(PO_4_,SO_4_,CO_3_)6F_2−3_)] and colophane (Ca_5_(PO_4_,CO_3_)_3_F, also spelled collophane)^2,3^. Geochemical studies can help understand the elemental composition and distribution of trace elements in phosphate deposits.

**Fig. 1:**
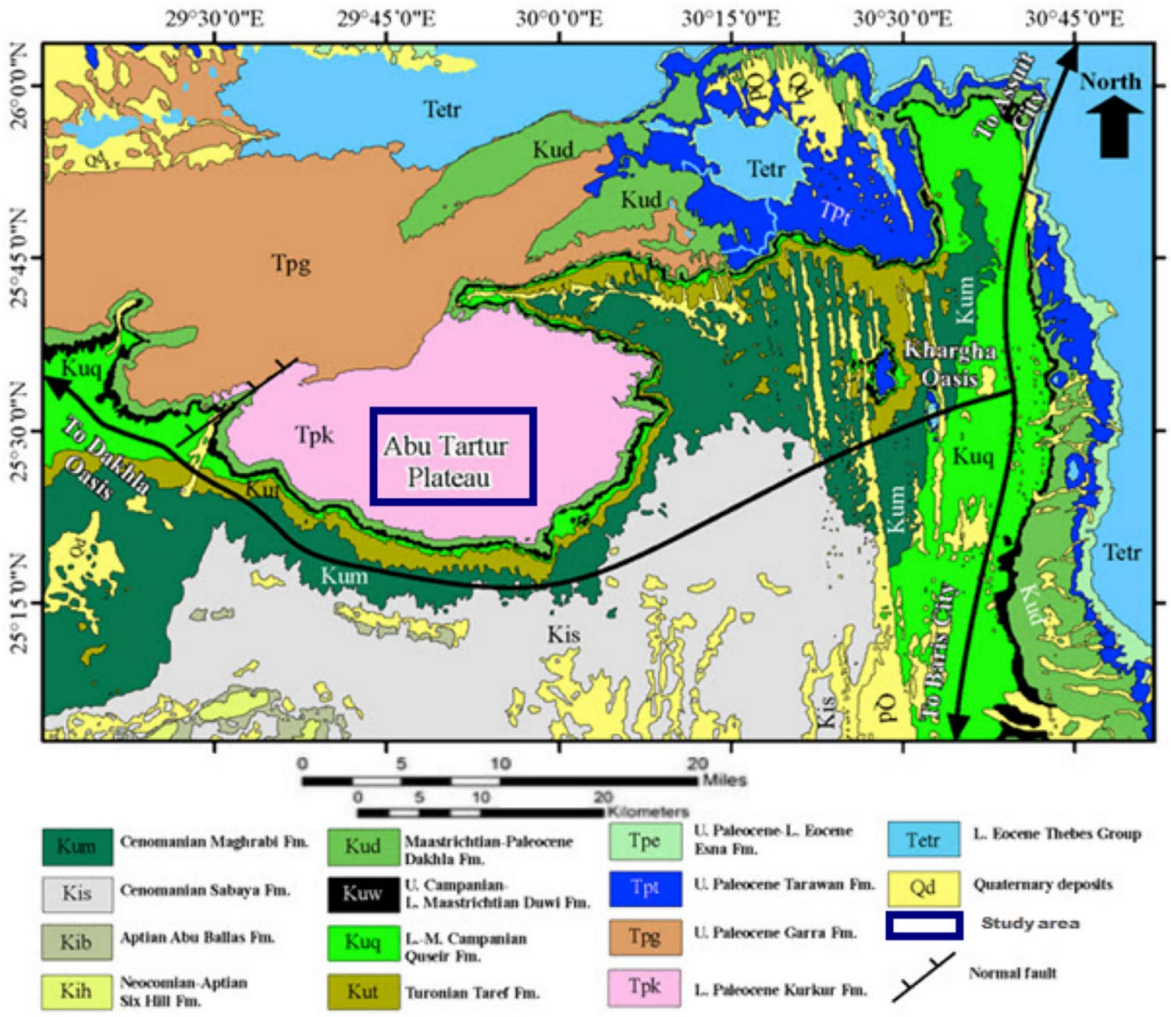
Displays the Geological map of Abu Tartur Plateau area Egypt, modified from the geological map of Egypt (modified after Conoco, 1987).

Phosphorrite beds are extracted from Duwi Formation by mining operations in Abu Tartur (Fig. 1), which shows redisposition and reworking, and is inter-bedded with black shales. Glauconite facies are a characteristic of Duwi Formation that grew gradually during the Late Cretaceous. The transition from shallow to deep marine conditions is the main feature of the contact between the Duwi and Dakhla Formations.

Phosphate minerals have essential electrical conductivity and ionic properties that vary with temperature. There are few investigations on the effects of temperature and electrical properties on natural phosphate mixtures^4,5^. Abdel-Kader^4^ establish that electrical conductivity from Abu Tartur of natural apatite increases with temperature, indicating a thermally activated conduction process while El-Shahat^5^ stated that the electrical conductivity of phosphate minerals also increases with temperature, proposing comparable thermal activation performance,. Due to larger charge carrier movement, both studies establish how high temperatures increase electrical conductivity.

Temperature variations impact mineral electrical behavior, composition, and structure, which may impact electrical conductivity. Understanding how temperature influences phosphate rocks’ electrical properties is essential to maximize mineral processing and geophysical exploration approaches. Electrical characteristic measurements can aid geophysical exploration techniques in defining ore zones and determining mineralization levels.

The temperature can enhance several actions that significantly influence the electrical conductivity of Phosphate rocks (e. g. thermal ionization, excitation, and hopping mechanisms). Phosphate rocks naturally display low electrical conductivity at room temperatures; though, their conductivity increases significantly at higher temperatures. This obvious performance is mainly recognized to improve ion mobility and the thermal activation of charge carriers as the temperature increases. In this study, the electrical properties of phosphate materials were analytically explored over a broad range of temperatures and frequencies, providing valuable considerations into the original conduction mechanisms. The unique temperature and frequency range used in this research is what makes it scientifically innovative, as well as the new insights into conduction mechanisms and electrical properties of natural phosphate samples.

## Egypt’s principal phosphate-bearing regions

Abu Tartur Plateau covers an area of about 1,000 km^2^. The surface of it is either gently sloping or flat, and the average height is ~ 300 m. The area has phosphate deposits, which are found in sedimentary and magmatic rocks, and it has a flat to moderately sloped surface. The phosphate deposits near Abu Tartur have a large amount of minerals, with fluorapatite, calcite, and quartz being the most prominent ones; however, some dolomite, pyrite, calcite, and iron oxides are also present^4^. The deposits have been subject to considerable weathering and modification, leading to the formation of complex mineral accumulations^6,7^.

During the Late Cretaceous period, phosphore-rich sediments were made up of the southern Neo-Tethys epicontinental shelf^5,8,9^. Duwi Formation contains phosphate layers with Quseir shale underneath and Dakhla shale above. Total rare earth elements (REEs) level in the phosphate rock is ~ 1810 ppm (~ 0.18%). Abu Tartur phosphate mine produces both liquid and solid wastes as a result of its mining and enrichment processes^10–12^. Waste rock stock piles, residues, and slurry primarily made of sand and clays are examples of solid wastes. The liquid wastes produced during the enrichment process are stored in residue ponds. Because of the heavy metals, radioactive materials, and tiny particulate matter included in these wastes, has a negative effects on the environment and human health.

A. *Abu Tartur Plateau (Western Desert):* Abu Tartur plateau’s phosphate deposits are part of Duwi Formation^13,14^. In succession, thick beds of phosphorite are often interbedded with shale and marl that contain a lot of organic matter. Usually, the phosphorite layers have a thickness of 1-3 meters.
B. *The Duwi and Quseir formations:* Are represented in the deposits at Safaga-Quseir Area (Eastern Desert). Shale and limestone intercalate the phosphate layers; the thickness of individual phosphate beds varies from a few centimeters to several meters^15^.
C. *Red Sea Coast*: Duwi Formation deposits are present in this region due to tectonic activity related to the Red Sea Rift. The presence of phosphate deposits is present in marine shales and limestones^16^. The thickness and quality of the phosphate beds vary considerably^17^.
D. *Nile Valley:* Quseir Formation is located in tertiary environments that are more stable, in comparison to the Red Sea coast. The interaction between shale and limestone with phosphate-bearing strata creates layers that are relatively continuous.

Uranium and other valuable elements, including lanthanides, are likely to be sourced from Abu Tartur phosphate deposit in Egypt’s Western Desert. Figure. 1 shows the Geological Map and Location of Natural Phosphate in Egypt.

## Geological settings and mineralization

The tectonic history of the area is linked to the structural evolution of Egypt’s phosphate reserves. The Western Desert is affected by several tectonic events, such as the collision between the Africans and Arabs, the Red Sea rifting, and the growth of the Mediterranean Sea. The occurrence of these events led to the formation of multiple faults, folds, and fractures, which in turn regulated the distribution and deposition of the phosphate deposits.

Duwi and Quseir Formations are the primary phosphate-bearing formations that were formed during Upper Quaternary to Paleogene sedimentary era^18,19^. This is a thorough synopsis of these formations:

1. *Duwi Formation (*Campanian to Maastrichtian era of Upper Cretaceous): it ismainly shale, marls, limestone, and phosphorites. Phosphate deposits are inter-bedded with shale, marl, and occasionally limestone. Its thickness varies widely (up to 70 m). It shows marine depositional environment with extensive upwelling zones.
2. *The Upper Cretaceous to Lower Paleogene Quseir Formation:* It shares lithology with Duwi Formation. Quseir Formation (~50m thickness) is made up of shale, limestone, and phosphate rock. Phosphorite minerals were deposited in shallow marine environments^20,21^.

The structural geology of Abu Tartur plateau is attributed to the flat-lying sedimentary layers. Minor folding (wider structural regime) and faulting (connected to the region’s extensional tectonics since the Mesozoic era), which are typical of the Western Desert’s larger tectonic framework.

The Surrounding environment facilitates the fine-grained deposition of particles, including phosphate minerals^22^. Two sub-environments comprise the shallow marine environment [*Inner shelf:* characterized by shallow, quiet waters (deposition of phosphate fine-grained minerals, e. g. calcite and fluorapatite); and *Outer shelf* (deposition of coarser grains phosphate, e. g. iron oxides and quartz)].

The geological background and location of Egypt’s phosphate deposits determine its composition. However, common deposits are composed similarly, with: A) P_2_O_5_ (20% to 35%); B) CaO (40% to 55%); C) Fe_2_O_3_ (2% to 10%); D) SiO_2_ (5% to 20%).

The geochemical properties of phosphate deposits are (1. Rare earth element (REE); (2. U and Th contents; and (3. Sr and Ba contents.

The following are the natural phosphate minerals compositions: Apatite (Ca_5_(PO_4_)_3_(F, Cl, OH)); Manganite ((Ce, La, Nd, Th) PO_4_); Xenotime (YPO_4_); Turquoise (CuA_l6_ (PO_4_)_4_ (OH)_8_ 4H_2_O); Variscite (AlPO_4_.2H_2_O).

Most of phosphate minerals exhibit complex compositions with altering elemental amounts, as Table 1 demonstrates^20,21^.

**Table 1:** summarizing the average concentrations (%) of elements at natural phosphate samples.

Mineral	Ca	P	O	F/Cl/OH	Ce	La	Nd	Th	Y	Cu	Al	H_2_O
Apatite	39–42	18–19	38–40	3–4	-	-	-	-	-	-	-	-
Monazite	-	13–14	30–35	-	27–30	12–15	10–12	5–10	-	-	-	-
Xenotime	-	19–20	45–46	-	-	-	-	-	34–35	-	-	-
Turquoise	-	9–10	36–38	-	-	-	-	-	-	6–8	30–31	9–10
Variscite	-	19–20	48–50	-	-	-	-	-	-	-	22–23	8–9

The main components are: *Limestones* (> 70%); calcium carbonate (CaCO_3_)); *Dolomites* (CaMg(CO_3_)_2_, ~ 20%; *Sandstones* (~ 5%); *Shales* (~ 5%). Clay minerals make up the majority of the shales’ composition and are often fossiliferous.

The Lithology arrangement of sedimentary rocks at Abu Tartur Plateau:

1. *Phosphorite:* Fluorapatite is frequently found in conjunction with dolomite and limestone. Phosphorites generally have a granular structure, and the phosphate grains are frequently held in place by a dolomite or calcite matrix^23,24^. The grains may have a nodule, pelletal, or oolitic shape. Phosphorites are frequently found in conjunction with quartz, clay minerals kaolinite and illite, and organic materials.
2. *The limestone-dolomite sequence:* It is made of calcite, which contains pieces of fossils, and it could show signs of secondary dolomitization^25,26^. There are fine-grained micrites and coarser-grained sparites among the limestones.
3. *Sandstone-shale sequence:* It is rich in organic materials, clay minerals, and different concentrations of carbonate. While marls have a more varied texture with fine and coarser components, shales are usually finely grained.

## Materials and methodology

### Electrical techniques

For the electrical testing, thin compact disks with a diameter to thickness ratio larger than 5 to 1 were used. This is used to minimize mistakes caused by stray capacitance^27^. The sample surfaces were polished to be parallel. We used Hitester Impedance Analyzer (Hioki, 3522–50 LCR). Samples measured between 42 Hz and 5 MHz frequencies and it were attached to non-polarizing electrodes (Cu/CuSO_4_)^28^.

Either a parallel or series configuration can be used to describe the electrical properties of a substance. You may find further details on the equations used, along with the factors that were measured, in^29,30^). The measurements were done in a closed area (a desiccator) with a relative humidity of 47% and an outside temperature of 31 °C. The electrode impedance was found to be less than 15 ohms within the tested frequency range^31–33^. Samples were gathered from various plateau locations. It was then carefully blended (to be homogeneous). In an effort to ensure that the samples are as representative as possible, they were taken from the majority of plateau regions. After that, it was ready for electrical evaluation.

### Temperature and frequency effects on AC conductivity

The recovery of phosphate minerals can be enhanced by improving mineral processing methods like flotation and electrostatic separation by utilizing the temperature and frequency dependency of conductivity. Apatite (Ca_5_(PO_4_)_3_ (F, Cl, OH)), monazite ((Ce, La, Nd, Th) PO_4_), and other phosphate-containing minerals are examples of compounds that are commonly found in phosphate natural mixes. These materials frequently have inherent electrical conductivity and ionic characteristics that are temperature-dependent and dependent on many other parameters (e. g. composition, grain size …)^34–38^.

Nevertheless, a shortage of studies on the influence of temperature and AC electrical conductivity on phosphate natural mixtures from Abu Tartur plateau persists. The intricate mineralogy and geochemistry of the phosphate deposits near Abu Tartur influence their electrical characteristics and behavior at varying textures, temperatures and frequencies^39,40^.

Understanding the electrical characteristics of phosphate rocks requires knowledge of AC electrical conductivity. It is a measurement of how easily an electric current moves through a substance. Temperature, porosity, and mineral composition all have an impact on AC electrical conductivity in all rocks. Phosphate rocks’ AC electrical conductivity is essential for a number of uses, such as environmental monitoring, mineral processing, and geophysical investigation^41^.

AC electrical measurements demand putting the material under alternating current (AC) and monitoring the voltage and current that results. The electrical conductivity and dielectric characteristics of the material can all show much information from the AC electrical tests. AC electrical measurements find extensive and varied applications in fields such as materials science, biomedicine, and geophysics^30,42,43^. In particular, electrical conductivity is a crucial property for investigating subsurface features. Measurements of electrical conductivity can help with geophysical exploration techniques to define ore zones and determine the amount of mineralization. There exist many methods for measuring the AC electrical characteristics of materials, such as: (*1. Dielectric spectroscopy:* dielectric characteristics^44,45^ as a function of frequency (dielectric loss, relaxation time, and dielectric constant); (*2. Impedance spectroscopy:*impedance^27^ in relation to frequency (electrical conductivity, impedance, and dielectric features); (*3. Conductivity* characteristics: (electrical conductivity and mobility)^24,40,46^

AC electrical conductivity of a phosphate natural combination from Abu Tartur and its fluctuation with temperature is inadequate and this work seeks to close this information gap. The porosity, mineral content, and AC electrical conductivity of the phosphate natural mixture will be ascertained by means of a combination of analytical methods and laboratory experiments in this^6,13,14,24^.

One class of minerals whose electrical conductivity is highly dependent on temperature is phosphate rocks. Depending on the features of the material and the temperature variety, the conductivity of the sample is changed as the temperature fluctuates. The relationship between conductivity and temperature is critical for multiple applications (semiconductors and minerals processing). Classically, the temperature requirement (Arrhenius equation) of a material’s conductivity (σ) is described by:

$$\sigma \left( T \right) = \sigma_{0} \exp \left( {{{E_{0} } \mathord{\left/ {\vphantom {{E_{0} } {kT}}} \right. \kern-0pt} {kT}}} \right)e$$

where $k$ is the Boltzmann^47^ constant (1.380694 × 10^–23^ J/K), $E_{0}$ is the activation energy (kJ/mol) (the energy barrier that charge carriers must cross to contribute to conduction), $\sigma_{0}$ the pre-exponential factor and $\sigma \left( T \right)$ is the electrical conductivity at absolute temperature $T$ (in Kelvin). The activation energy $E_{0}$ can be established from plotting ln(σ) versus ${1 \mathord{\left/ {\vphantom {1 T}} \right. \kern-0pt} T}$ and calculating the slope of the resulting line. As the temperature increases, the dielectric loss peaks change in the direction of higher frequencies owing to a decrease in dielectric relaxation periods. AC electrical conductivity is temperature dependent and involves complex interactions between electronic and ionic conduction pathways.

Certain materials (e. g. ceramic materials containing barium titanate) have a positive temperature coefficient (PTC), which means that as the temperature rises, so does their conductivity^48,49^. In certain semiconductors and metals, this phenomenon is frequently seen. The enhanced conductivity and mobility of the charge carriers, arising from their higher thermal energy, is frequently attributed to the PTC. Certain substances have a negative temperature coefficient (NTC), indicating that their conductivity decreases with temperature rise. This kind of action is commonly observed in some insulators and semiconductors^50^. Generally, the NTC is ascribed to the charge carriers’ higher thermal energy, which raises their rate of scattering and lowers their mobility and conductivity. Additionally, temperature can activate a number of processes that influence a material’s conductivity, such as: (*1. Thermal ionization:* Heat energy ionize impurities and increase charge carriers quantity and conductivity; (*2. Thermal excitation:* Charge carriers are excited and move from valence bands to conduction bands and increase charge carriers quantity and conductivity; (*3. Thermal hopping:* charge carriers hopping between localized states is facilitated by thermal energy, which raises conductivity.

The conductivity of phosphate rocks, such as those in the Abu Tartour Plateau, varies intricately with temperature. Phosphate rocks normally have a low conductivity at ordinary temperature, but at high temperatures, that conductivity can rise dramatically. The enhanced mobility of ions at high temperatures and the thermal activation of charge carriers are responsible for this behavior. Increased phonons (lattice vibrations) at higher temperatures can either help or delay ion migration by scattering ions or by supplying energy to overcome potential barriers. AC electrical conductivity is significantly impacted by frequency as well. At low frequencies, conductivity is argued to space charge areas and electrode polarization. Because of the increased ion mobility caused by temperature changes, these low-frequency responses can be greatly impacted. The bulk characteristics of the material dominate the response at higher frequencies. More noticeable are the dielectric relaxation processes and intrinsic electronic characteristics^51,52^.

A higher temperature increases conductivity. The electrical properties of materials can be significantly impacted by variations in heterogeneity^53^. Although these characteristics are mostly dependent on conductor concentration, differences prevents them from rising steadily as concentration increases. Understanding electrical properties and geological features is significantly impacted by analysis of temperature and heterogeneity. This approach clarifies the complicated connection between temperature, conductivity, and the consequences of heterogeneity created by additional variables such changes in material composition.

Conductivity rises with ion concentration because the movement of ions in solution causes temperature to rise as dissolved ionic substances does^47^. Electricity can pass through materials more readily at greater temperatures. The barrier hopping process can be used to explain how conductivity increases with temperature. The hopping of charge carriers between localized states is the cause of the temperature-dependent increase in conductivity.

A power law, σ_ac_ = Aω^n^^47^, represents the Jonscher’s universal law, where σ_ac_ is the AC conductivity, A is a constant, n is a fractional exponent (< 1), ω is the angular frequency (2 $\pi$ f), f is the frequency. The conductivity’s temperature dependency suggests that thermal activation is the process that causes electrical conduction.

We shall attempt to address how temperature variations may impact electrical qualities in this research. Making certain electrical properties for various phosphate samples is our goal. Different temperatures and frequency ranges were investigated for electrical properties. Phosphate rocks’ electrical characteristics are mostly determined by temperature and frequency. Generally, rock types, fluid conduction, metallic and semiconductor electron conduction, contained fluid, fluid saturation level, porosity and permeability, pore connectivity, chemical inhomogeneities, depositional or crystallization conditions, temperature, and component orientation can all have an impact on a rock’s electrical conductivity^54,55^.

## Results

**Fig. 2:**
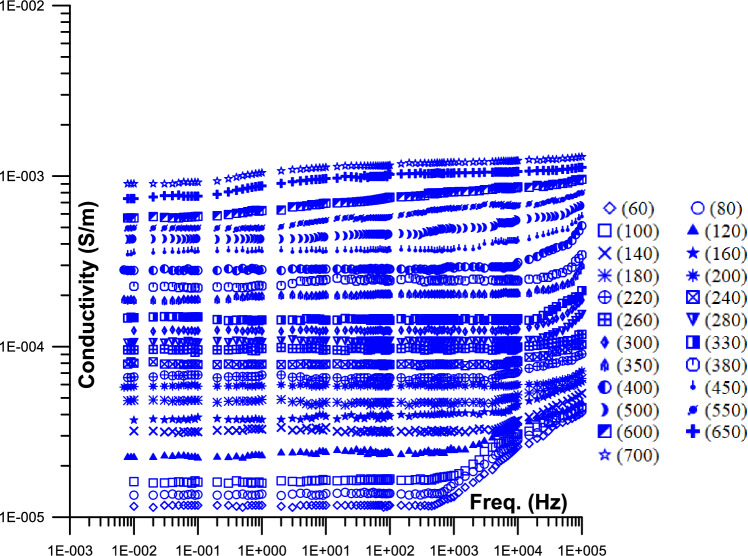
Displays the 2D conductivity of phosphate samples against frequency as a function of different temperatures (from 60 to 700 °C); [ (60),  (80),  (100),  (120),  (140),  (160),  (180),  (200),  (220),  (240),  (260),  (280),  (300),  (330),  (350),  (380),  (400),  (450),  (500),  (550),  (600),  (650),  (700)].

Figure 2 displayed the conductivity of phosphate samples against frequency as a function of temperature (from 60 to 700 °C). As the frequency increases, the conductivity slope starts very gentle up to 700 Hz at a specific temperature (it varies depending on the temperature), after which it starts to grow monotonically. The conductivity value is 10^–5^ S/m (42 Hz) at 60 °C and 10^–3^ S/m (42 Hz) at 700 °C temperatures. At 60 °C, the conductivity value is 3X10^–5^ S/m (100 kHz), while at 700 °C, it is 10^–3^ S/m (100 kHz). At various temperature levels, the conductivity with frequency exhibits a power law $\sigma \propto w^{m}$ characteristic with rising conductivity with frequency.

Figure 2 Displays the 2D conductivity of phosphate samples against frequency as a function of different temperatures (from 60 to 700 °C).

**Fig. 3:**
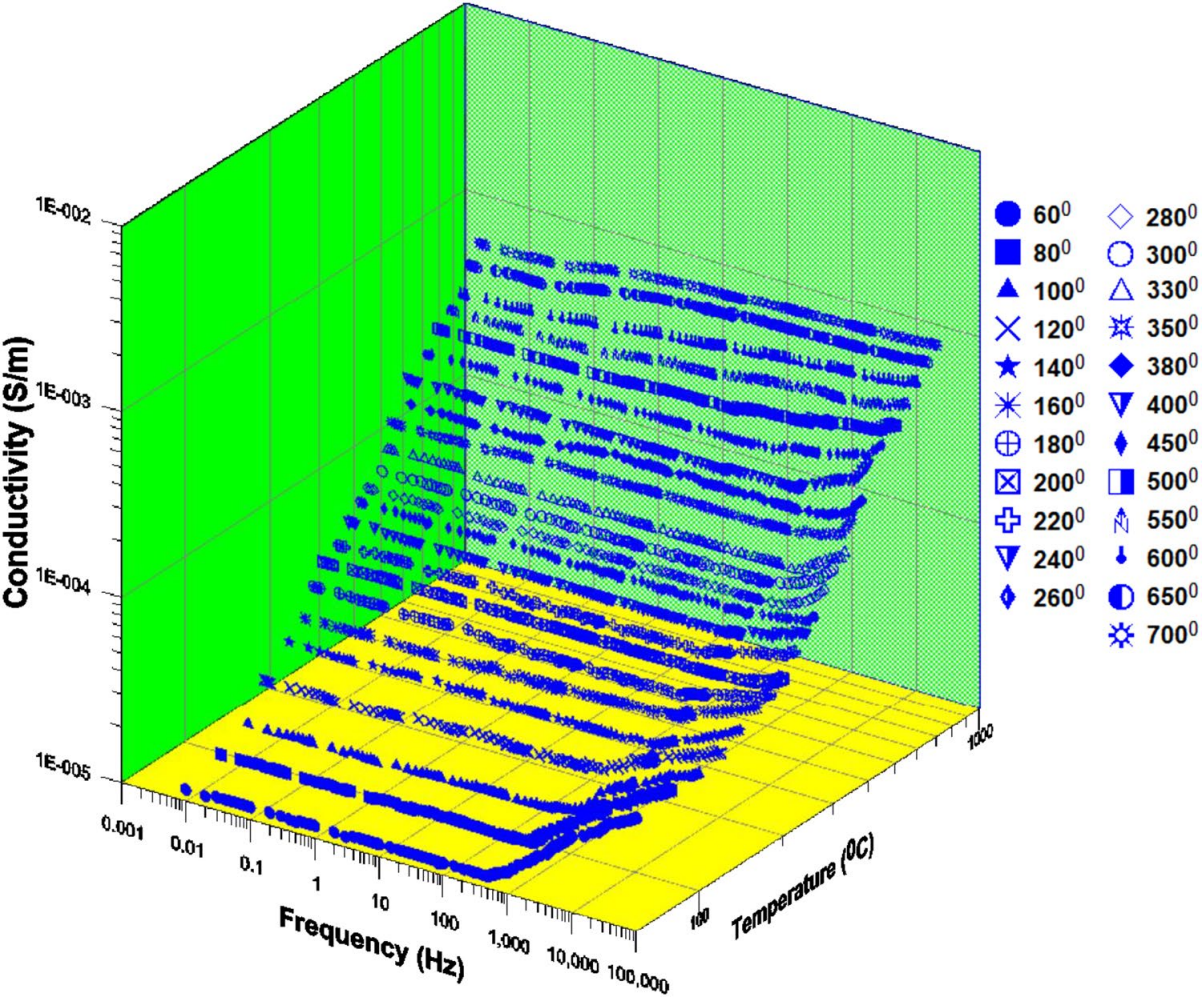
Displays the 3D conductivity of phosphate samples against frequency as a function of different temperatures (from 60 to 700 °C); [ (60),  (80),  (100),  (120),  (140),  (160),  (180),  (200),  (220),  (240),  (260),  (280),  (300),  (330),  (350),  (380),  (400),  (450),  (500),  (550),  (600),  (650),  (700)].

Figure 3 Displays the 3D conductivity of phosphate samples against frequency as a function of different temperatures (from 60 to 700 °C). The value of the dielectric constant at 60 °C is 5X10^6^ (42 Hz), and at 700 °C it is 6X10^8^ (42 Hz). At 60 °C, the conductivity value is 15 (100 kHz), while the dielectric constant value is 90 (100 kHz). The dielectric constant often exhibits a power law dependency $\varepsilon ^{\prime} \propto f^{n}$ with an exponent of n $\approx$−0.55 (Jonscher^56,57^. The dielectric constant slope steeply decreases with increasing frequency up to 700 Hz at a particular temperature (which varies)^58^. After that, it begins to settle down with a fairly gentle slope^45,59^. As the temperature rises (from 700 Hz to higher values), the frequency value of the knee at which the slope shifted from moderate to acute is increased (significantly).

**Fig. 4:**
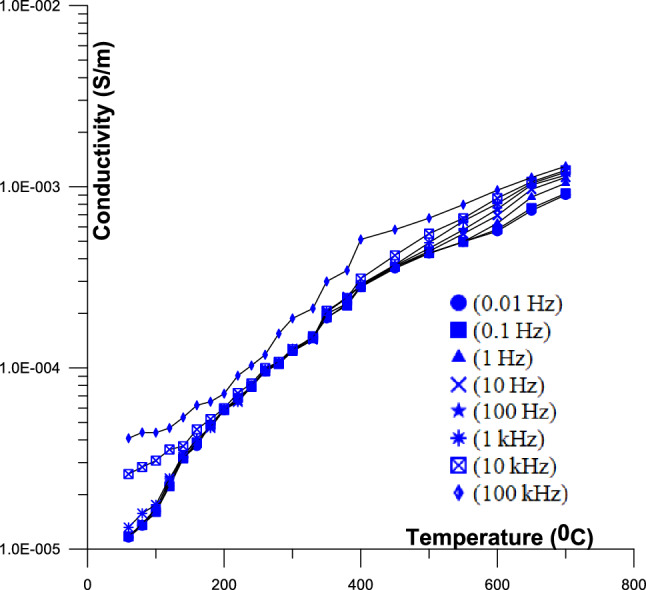
Displays the 2D conductivity of phosphate samples against temperatures as a function of different frequency (from 60 to 700 °C); [ (0.01 Hz),  (0.1 Hz),  (1 Hz),  (10 Hz),  (100 Hz),  (1 kHz),  (10 kHz),  (100 kHz)].

Figure 4 Displays the 2D conductivity of phosphate samples against temperatures as a function of different frequency (from 60 to 700 °C).

**Fig. 5:**
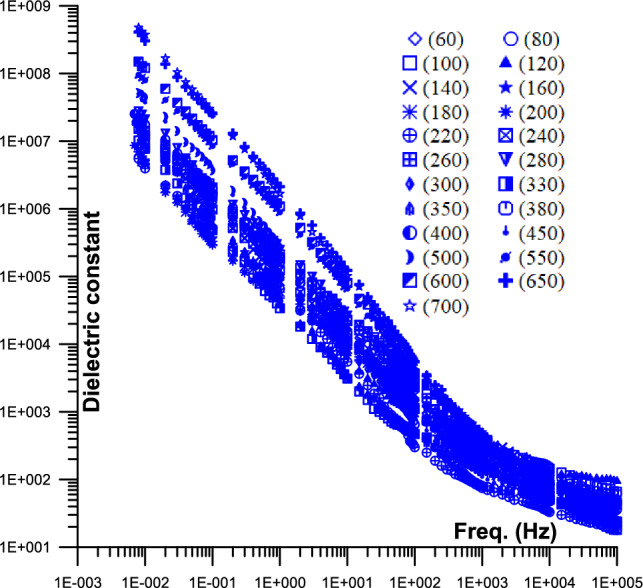
Displays the 2D dielectric constant of phosphate samples against frequency as a function of different temperatures (from 60 to 700 °C); [ (60),  (80),  (100),  (120),  (140),  (160),  (180),  (200),  (220),  (240),  (260),  (280),  (300),  (330),  (350),  (380),  (400),  (450),  (500),  (550),  (600),  (650),  (700)].

Figure 5 Displays the 2D dielectric constant of phosphate samples against frequency as a function of different temperatures (from 60 to 700 °C).

**Fig. 6:**
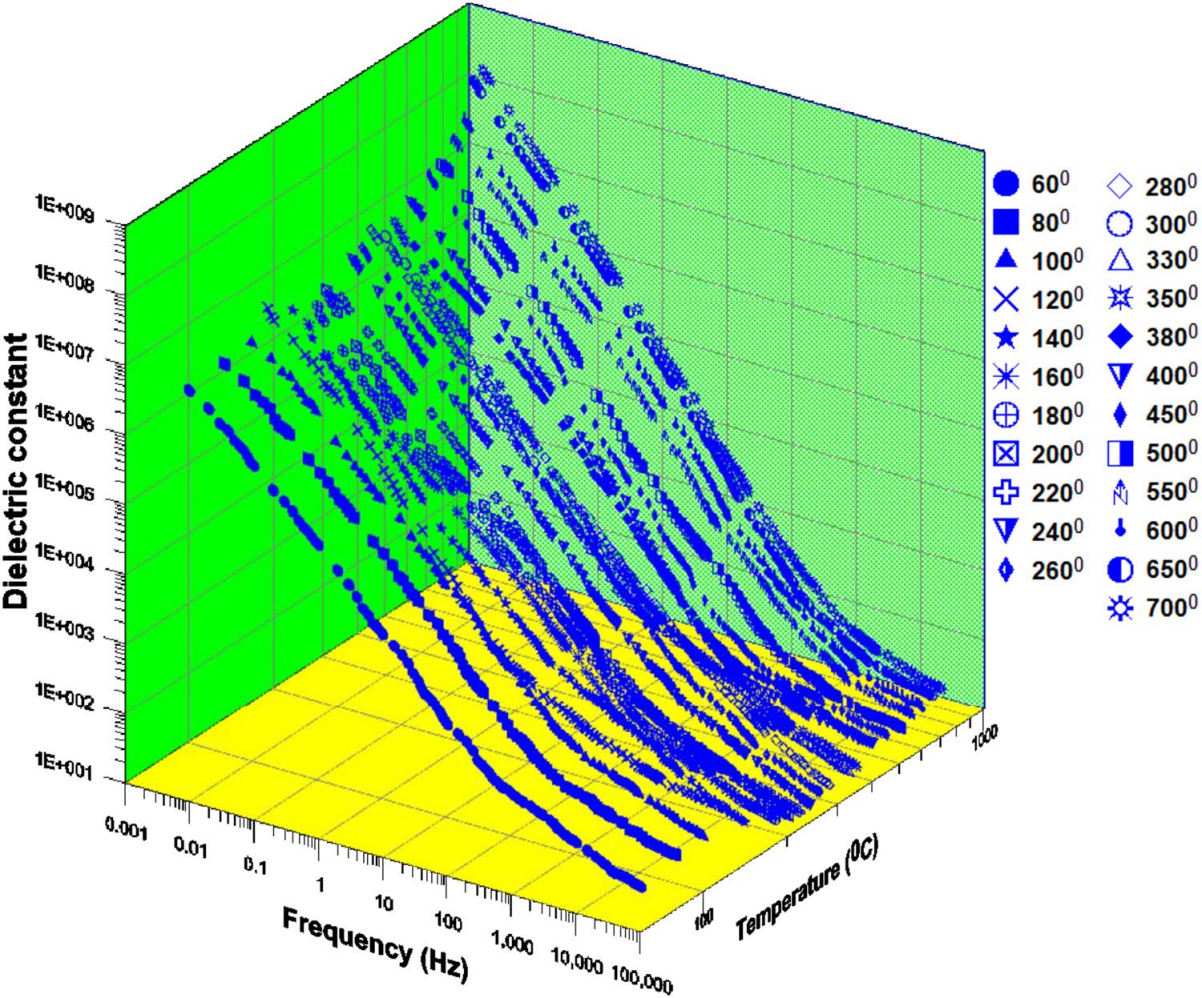
Displays the 3D dielectric constant of phosphate samples against frequency as a function of different temperatures (from 60 to 700 °C); [ (60),  (80),  (100),  (120),  (140),  (160),  (180),  (200),  (220),  (240),  (260),  (280),  (300),  (330),  (350),  (380),  (400),  (450),  (500),  (550),  (600),  (650),  (700)].

Figure 6 Displays the 3D dielectric constant of phosphate samples against frequency as a function of different temperatures (from 60 to 700 °C). The number of conductor elements found in each specimen allows for the distinction of two sets of specimens. The first one is composed of rich clay or carbontes (and occasionally water) and has a very high concentration of clay (very little sand). This group’s conductivity ranges from 10^–2^ to 10^–1^ (S/m). The second sample set has a low percentage of clay and a comparatively large quantity of sand. This group’s conductivity ranges from 10^–2^ to 10^–3^ (S/m). High amounts of water are present in samples with conductivity values greater than 10^–1^^60–63^.

**Fig. 7:**
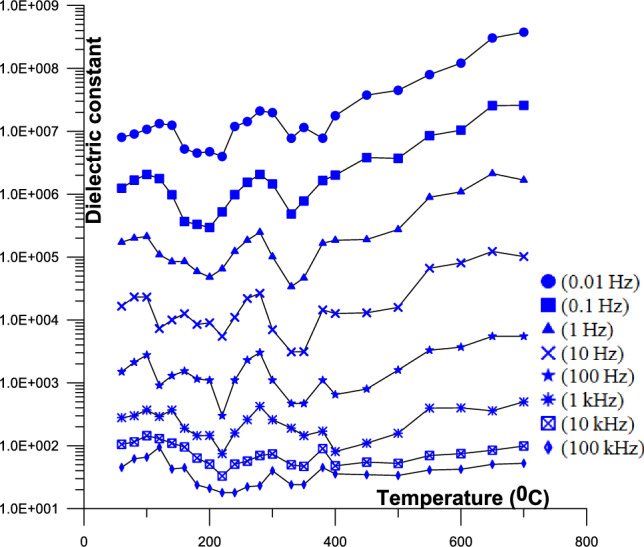
Displays the 2D dielectric constant of phosphate samples against temperatures as a function of different frequency (from 60 to 700 °C); [ (0.01 Hz),  (0.1 Hz),  (1 Hz),  (10 Hz),  (100 Hz),  (1 kHz),  (10 kHz),  (100 kHz)].

Figure 7 Displays the 2D dielectric constant of phosphate samples against temperatures as a function of different frequency (from 60 to 700 °C). Three sets of specimens can be distinguished based on the quantity of broken pathways that exist between two insulator clusters in the specimens. The first set of curves has a single slope. For the second set of curves, there are two slopes. The final set of curves has a frequency-flat response. The first group contains an exceptionally high concentration of isolated carbontes, or clusters of sand, and occasionally isolated water. The dielectric constant range for this group is 10^5^ to 5X10^7^ at low frequency (42 Hz). In comparison, the second group has a lesser concentration of isolated carbontes, isolated water, or isolated sand clusters. The dielectric constant for this group is between 6X10^7^ and 10^9^ (42 Hz). The third group has low amounts of carbontes, water clusters, or solitary sand. The dielectric constant range for this group is 7X10^1^ to 3X10^2^ (42 Hz).

## Discussions

Insulating grains block electrical pathways. The study’s findings demonstrate that there is significant temperature dependence in the AC electrical conductivity of the phosphate natural mixture from Abu Tartur plateau in Egypt’s Western Desert. The conductivity exhibits a positive temperature coefficient (PTC) characteristic as the temperature rises. Furthermore, with conductivity raising with frequency the conductivity’s frequency dependence is noted.

Figure. 2 displayed the conductivity of phosphate samples against frequency as a function of temperature (from 60 to 700 °C). The frequency value of the knee at which the slope changed from flat to frequency-dependent is increased with the increase of the temperature value (from 700 Hz to higher values; may be beyond the frequency limit of this study). The conductivity increases monotonically with temperature, and the conductivity abruptly increases (beyond 700 °C) because of the increase in continuous pathways, which is comparable to the conductivity behavior at a percolation threshold^64^. At high temperatures, the conductivity value is higher than the saturated samples’ typical values (Fig. 2). Increased activity levels and charge carrier hopping between localized states, which promotes thermal energy at high temperatures, could be the cause of this^65^.

At low frequencies, the moving surface charges polarize; at higher frequencies, the conductivity is increased because the cycle period is less than the time it takes for the charges to polarize^66^. The higher temperatures in Fig. 2 only exhibit a single, almost zero slopes with frequency, indicating that the majority of electrons are able to cross potential barriers. Few continuous conducting channels exist at low temperatures, and many conducting pathways are obstructed by air (most electrons are unable to pass over potential barriers)^67^. There are several unconnected conductor paths and high air capacitor resistance at low frequencies. The resistance of air capacitors decreases with increasing frequency, while associated conductor paths start to increase^33^.

Figure. 5 displayed the 2 D dielectric constant of phosphate samples against frequency as a function of temperature (from 60 to 700 °C). The behavior of the dielectric constant is due to the electrons’ capacity to cross potential barriers and increasing energy levels, as well as the decreasing distances between conducting grains, the dielectric constant decreases monotonically with temperature. Until the temperature crosses the percolation threshold, the dielectric constant decreases^68–70^.

The 3D dielectric constant as a function of temperature (from 60 to 700 °C) is plotted against frequency in Fig. 6. The dielectric constant’s power law variation with frequency is caused by the sample’s numerous pore throats and gaps between different conducting grains. According to^71^, as temperature raises, mobile surface charges raise as well, contributing to polarization and a decrease in the insulator distances between conducting grains^72,73^.

The study’s findings show that frequency and temperature have a significant impact on the phosphate natural mixture’s AC electrical conductivity^74,75^. The conductivity’s frequency dependence is explained by the charge carriers’ relaxation, which causes conductivity to rise at high frequencies^76–78^. The conductivity and dielectric constant decrease with frequency and increase with conductor material, saturation, and clay concentration^79,80^.

The dielectric constant is increased by the conductive percentage due to the appearance of clustering inclusions (decrease in the thickness of the insulating gaps between conductive clusters)^47,81–83^.

The electrical properties of mixes are often influenced by the composition of clay and the ionic concentration of the pore fluid, or salinity^84–89^.

The dielectric constant change with frequency for the Wadi Saal, East Central Sinai samples is shown in Fig. 6. Clay minerals have an impact on a mixture’s cation-exchange capacity; as a result, an increase in clay content is likely to cause an increase in the sample’s electrical conductivity^80,88,90–95^.

In general, when frequency and conductor concentration increase, more continuous conductor connections between grains and minerals begin to form. Because of the increased energy barriers between atoms brought on by rising particle activity levels, conductivity values rise with frequency^96–98^.

The samples curves with high conductivity (> 10^–1^ s/m) at low frequencies are thought to have a comparatively high total conductor concentration. The majority of the curve’s samples with relatively low conductivity values (< 10^–1^ s/m) at low frequencies also have relatively low total conductor concentrations and a single slope (0.2) that represents the frequency dispersion^44,56,99–101^.

The dielectric constant’s (Fig. 7) observed continuously increasing value with increasing concentration of total conductor can be explained by a capacitive effect caused by a decrease in the space between conductor grains^102^. Narrow insulating gaps between granules or clusters of semiconducting materials affect the high dielectric constant values^80,103–106^. The dispersion of pore openings is responsible for the frequency-dependent relationship between the dielectric constant and the sample, as^47,56^ and Gomaa and Elsayed^29^ have discussed.

The existence of the insulator and conductor components can have a substantial impact on the mixture’s dielectric qualities^35,107^.

The study’s findings will shed important light on the behavior of these phosphate deposits’ electrical conductivity, which will help determine how best to exploit and use them. The findings of this study may have implications for geophysical exploration, mineral processing, and environmental monitoring since they will shed light on the electrical characteristics of phosphate rocks from Abu Tartur and how they behave at various temperatures.

## Conclusion

Abu Tartur plateau petrography displays a complicated mixture of limestones, marls, shales, and phosphorites. The investigations were carried out using a range of temperatures (60–700 0 C) and frequencies (42 Hz–5 MHz). Complex interplay between ionic and electronic conduction pathways is the result of temperature and AC electrical conductivity in samples. Conductivity and the dielectric constant both rise with temperature and frequency. The conductivity’s frequency dependence is explained by the charge carriers’ relaxation, which causes conductivity to rise at high frequencies. This implies that the phosphate combination has several relaxation times and a complicated impedance spectrum. Petrography show that samples is mostly composed of sedimentary rocks, with small amounts of metamorphic and igneous rocks (sandstones, shales, limestone, and dolomites). Connected clusters are formed as the conductive components rises, and this reduces the amount of insulating gaps between conducting components. An increase in clay content is correlated with an increase in electrical conductivity. Electrical properties are influenced by cation-exchange, surface conductivity, salinity, and clay content. Defining connections between lithology and electrical characteristics, porosity, and permeability is of great importance for this research. This study proposes to enhance our research of the geological and geochemical mechanisms that contribute to the development of phosphate reserves as it offers additional insight into the electrical conductivity behavior of these deposits, which will be helpful in identifying the most efficient methods to mine and utilize them. Additional investigation may yield additional understanding of the complicated relationship among temperature, electrical conductivity, and mineralogy.

## Data Availability

The datasets used and/or analyzed during the current study available from the corresponding author on reasonable request.
