# Correction: Temperature and AC electrical properties effects on phosphate natural mixture, Abu Tartur plateau, Western Desert, Egypt

**DOI:** 10.1038/s41598-025-23125-5

**Published:** 2025-10-08

**Authors:** Mohamed Mahmoud Gomaa

**Affiliations:** Geophysical Sciences Dept, National Research Centre, Geophysics Lab, Dokki, Cairo, Egypt

Correction to: *Scientific Reports* 10.1038/s41598-025-09313-3, published online 31 July 2025

In the original version of this Article part of the title was erroneously omitted.

“Temperature and AC electrical properties natural mixture, Abu Tartur plateau, Western Desert, Egypt”

now reads:

"Temperature and AC electrical properties effects on phosphate natural mixture, Abu Tartur plateau, Western Desert, Egypt"

The original Article has been corrected.

